# A 3D Attitude Estimation Method Based on Attitude Angular Partial Feedback for Polarization-Based Integrated Navigation System

**DOI:** 10.3390/s22030710

**Published:** 2022-01-18

**Authors:** Pengwei Hu, Panpan Huang, Zhenbing Qiu, Jian Yang, Xin Liu

**Affiliations:** 1 School of Automation Science and Electrical Engineering, Beihang University, Beijing 100191, China; hpw00@buaa.edu.cn (P.H.); jyang_buaa@buaa.edu.cn (J.Y.); xliubuaa@buaa.edu.cn (X.L.); 2 Hangzhou Innovation Institute, Beihang University, Hangzhou 310051, China; qiuzhenbing@buaa.edu.cn; 3 Beijing Advanced Innovation Center for Big Data-Based Precision Medicine, School of Medicine and Engineering, Beihang University, Beijing 100191, China

**Keywords:** polarization navigation, the solar vector, 3D attitude estimation, partial feedback

## Abstract

Polarization (POL) navigation is inspired by insects’ behavior of precepting celestial polarization patterns to orient themselves. It has the advantages of being autonomous and having no accumulative error, which allows it to be used to correct the errors of the inertial navigation system (INS). The integrated navigation system of the POL-based solar vector with INS is capable of 3D attitude determination. However, the commonly used POL-based integrated navigation system generally implements the attitude update procedure without considering the performance difference with different magnitudes of the angles between the solar-vector and body-axes of the platform (S-B angles). When one of the S-B angles is small enough, the estimated accuracy of the attitude angle by the INS/POL is worse than that of the strapdown inertial navigation system. To minimize the negative impact of POL in this situation, an attitude angular adaptive partial feedback method is proposed. The S-B angles are used to construct a partial feedback factor matrix to adaptively adjust the degree of error correction for INS. The results of simulation and real-world experiments demonstrate that the proposed method can improve the accuracy of 3D attitude estimation compared with the conventional all-feedback method for small S-B angles especially for yaw angle estimation.

## 1. Introduction

Polarization (POL) navigation inspired by insects has been widely investigated due to its full-autonomy and lack of risk of cumulative errors. The celestial polarization pattern (CPP) is formed under the effect of Rayleigh scattering of the atmosphere on the unpolarized sunlight. It contains geospatial information of the sun, the predictable feature of which shows the potential for serving as a natural compass. Many animals can detect the CPP to orient themselves [1,2]. The bioinspired POL navigation can be used to realize heading estimation [3,4,5] and full-autonomous positioning aided by other navigation methods [6,7,8].

However, the navigational information provided by CPP is incomplete. Generally, only the yaw angle is obtained from polarization measurements. It has been proved that the Rayleigh polarization model cannot determine 3D attitude alone in real-time [9]. To provide more polarization navigation, generally, the POL navigation is often integrated with an inertial navigation system (INS) to correct the errors of INS [10,11]. There are three commonly used strategies for integrating POL navigation with INS, which are briefly listed in Table 1.

In the first strategy, the angle of polarization (AoP) obtained by a monocular polarization unit is used to calculate the E-vector in the direction of the observed point on the celestial sphere. Subsequently, the E-vector is used for measurement modeling based on the vertical relationship with the solar vector. Although the E-vector is a 3D unit vector in form, the three elements of the vector are calculated from a single scalar, i.e., AoP. So the navigation information contained in one E-vector is limited. In the second strategy, the yaw angle is calculated by POL measurements aided by extra navigation means, such as inertial device for horizontal angles. Therefore, POL is often used to aid INS only for yaw estimation for the first two integrated strategies. Although the POL cannot determinate 3D attitude individually, the third strategy of the solar vector-based POL navigation can aid INS for 3D attitude angles estimation. It is due to the two uncorrelated scalars, the solar azimuth and zenith, contained in the solar vector that provides more navigation information. Herein, the third strategy of the solar vector-based integrated model is adopted.

**Figure 1:**
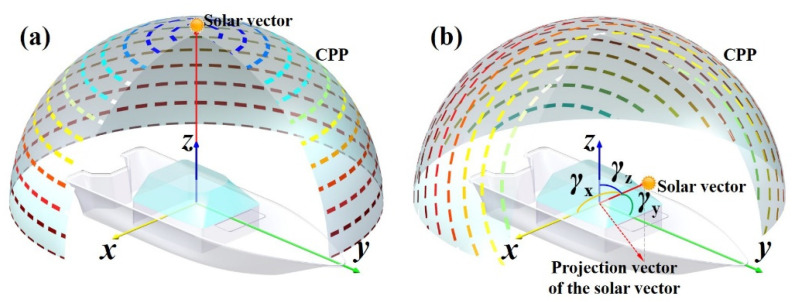
Spatial relationship between CPP, the solar vector and platform body axes when the sun (denoted as origin dot) is on the zenith point (**a**) and is nor near the zenith (**b**). The yellow, green, and blue arrows are body axes of the platform. The colored lines on the hemisphere represent E-vectors.

Regarding the navigation mechanism, the third strategy of the solar vector-based integrated model is similar to that of the celestial navigation model based on starlight vector integrated with INS. Generally, multiple stars are used for 3D attitude determination [21,22]. Different from the multiple-star-based celestial navigation, only one solar vector can be obtained in the POL navigation. The single-star-based celestial navigation integrated with INS was investigated [23,24]. One limitation of the integrated navigation model is the low accuracies 3D attitude determination which need to be improved by external navigation sensors. It can be illustrated by an extreme case when the sun is on the zenith point. The single-scattering Rayleigh polarization model cannot be used to yaw determination as shown in Figure 1a. In this extreme case, POL cannot aid INS for yaw estimation in the third integrated strategy. As shown in Figure 1b, the angles between the solar-vector and the body-axes of the platform, i.e., axis x, y, and z, (S-B angles) denoted as $\gamma_{x}$, $\gamma_{y}$, and $\gamma_{z}$, respectively. In this extreme case, $\gamma_{z}$ is nearly zero. Accordingly, a hypothesis is put forward that there is a relationship between $\gamma_{z}$ and the yaw correlation ability of POL for INS.

The yaw correction capability of the POL-based solar vector is considered to originate from the projection vector of the solar vector (as shown in Figure 1b) on the XOY-plane in the body frame (b-frame). The relative rotation according to the *z*-axis between the projection vector and the vehicle provides the change of yaw, which is used to correct the drifting errors of INS. In subsequence, the angle error of the projection vector affects the yaw estimation accuracy directly. Under a constant measurement error of each component of the solar vector, the angle error of the projection vector is influenced by the length of the projection that depends on the angle between the solar vector and the *z*-axis, i.e., $\gamma_{z}$. A large $\gamma_{z}$ leads to a long projection that further contributes improving the yaw estimation accuracy. On the contrary, when the sun is near the zenith, the projection decreases and approaches zero (see Figure 1a), which impairs the accuracy of the projection vector. Then the correction capability of POL is decreased. More generally, this phenomenon also exists in the estimation of two horizontal attitude angles. For the 3D attitude determination, the S-B angles will possibly affect the corresponding attitude estimation accuracy. Then, minimizing the negative effect of polarization measurement with small S-B angles will improve the performance of INS/POL under varying scenarios.

In the conventional all-feedback method, some states of the INS/POL integrated navigation filter are fed back to INS completely to correct its drift errors [17,18]. When one of the S-B angles is small, low-accuracy projection vector results in inaccurate correction information, which eventually leads to an even larger error than that using INS alone. Therefore, in the condition of small S-B angles, the attitude estimation should not be fed back to INS fully. Inspired by a partial feedback principle to avoid the negative effect of severe serrated output by an all-feedback method in an inversed strapdown inertial navigation system (SINS) algorithm [25], an attitude angular partial feedback method is proposed to address this problem. Herein, to minimize the negative effect of POL in the condition of small S-B angles, the states of misalignment angles estimated by the filter are fed back to INS partially. The partial feedback strategy can be designed based on the magnitudes of the S-B angle.

In this work, the solar vector-based POL navigation integrated with INS is adopted for 3D attitude estimation. To improve the 3D attitude angles estimation accuracy in the situation of small S-B angles, an adaptive partial feedback (APF) method is proposed. S-B angles are used as the parameters of partial feedback factors to adjust the correction degree for INS. A series of simulations and real-world experiments were conducted to analyze the performance of the proposed method. The results demonstrate that in the condition of small S-B angles, the negative effect of POL measurement for INS/POL system is reduced, especially for yaw estimation.

The remainder of this paper is organized as follows: In Section 2, the solar vector-based INS/POL integrated navigation model is established. Section 3 analyzes the limitations of the conventional all-feedback method through a group of simulation experiments, based on which the adaptive partial feedback method is proposed. Then, a series of simulation experiments were conducted to validate the effectiveness of the proposed method in Section 4. Furthermore, the performance in the realistic scenario of the proposed method is evaluated based on a real-world experiment in Section 5. Finally, conclusions are drawn in Section 6.

For the convenience of the readers, the main acronyms used in the paper are described in Table 2:

## 2. INS/POL Integrated Navigation System Modeling

Both the all-feedback method and APF are based on the solar vector-based INS/POL integrated navigation model which can be used for 3D attitude determination. To obtain the solar vector based on POL, at least two polarization E-vectors from different directions are required. The solar vector is calculated based on a set of compound eye polarization sensor. It is equipped with IMU to integrate the bioinspired polarization-based attitude and heading reference system (PAHRS), which is used in this paper. The solar vector can be calculated with the polarization E-vectors measured from the compound eye units. In the following section, the overview of PAHRS and solar vector calculation are first introduced, followed by the analysis of the conventional all-feedback methods’ limits based on simulated experiments.

### 2.1. The Solar Vector Calculation Based on the Compound Eye Polarization Sensor

The compound eye polarization sensor is composed of nine monocular polarization units to acquire polarization information from nine different points on the celestial sphere. IMU is mounted inside PAHRS. The features of PAHRS are given in Table 3 (for more about PAHRS see Ref [26]).

**Figure 2:**
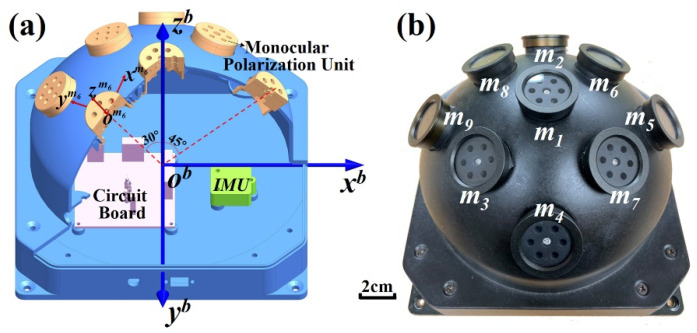
The section diagram (**a**) and photograph (**b**) of PAHRS.

The coordinate frames used in this work are defined as follows: the navigation frame (n-frame) which selects the geographic frame (E-N-U frame); the calculation navigation frame of INS (n’-frame); the body frame (b-frame) of PAHRS; and the model frame (m_i_-frame, i = 1, 2, …, 9) of each monocular polarization unit. The latter two frames are shown in Figure 2a.

The three attitude angles, pitch (θ), roll (φ), and yaw (ψ), are defined to form the attitude transfer matrix $C_{n}^{b}$, which represents the direction cosine matrix from n-frame and b-frame. The $C_{n}^{b}$ is expressed as $C_{n}^{b}=R_{x}\left(\theta \right)R_{y}\left(\varphi \right)R_{z}\left(\psi \right)$, where $R_{x}\left(\theta \right)$, $R_{y}\left(\varphi \right)$, and $R_{z}\left(\psi \right)$ [12] are the rotation matrix of pitch, roll, and yaw, which are shown as follows:

(1) $$R_{x}\left(\theta \right)=\left[\begin{array}{ccc} 1 & 0 & 0\\ 0 & \cos\theta & \sin\theta \\ 0 & -\sin\theta & \cos\theta \end{array}\right],R_{y}\left(\alpha \right)=\left[\begin{array}{ccc} \cos\alpha & 0 & -\sin\alpha \\ 0 & 1 & 0\\ \sin\alpha & 0 & \cos\alpha \end{array}\right],R_{z}\left(\psi \right)=\left[\begin{array}{ccc} \cos\psi & -\sin\psi & 0\\ \sin\psi & \cos\psi & 0\\ 0 & 0 & 1 \end{array}\right]$$

**Figure 3:**
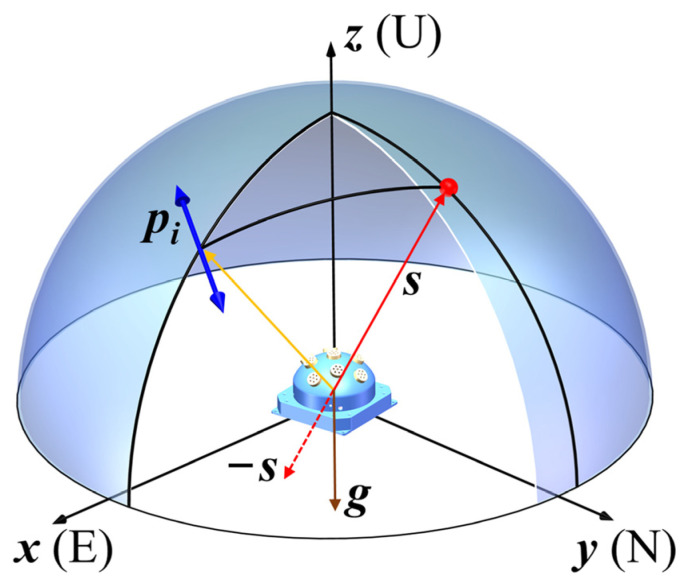
The geometrical description of the main vectors used in the solar vector calculation.

The solar vector in b-frame ($s^{b}$) calculated by the compound eye polarization sensor, is used to establish the measurement equation. Figure 3 shows the relationship of the main vectors used in solar vector calculation. The polarization angle measured by any monocular polarization unit is represented by $\alpha_{i}$. The E-vector in m**_i_** -frame can be described by

(2) $$p^{m_{i}}={\left[\begin{array}{ccc} \cos\alpha_{i} & \sin\alpha_{i} & 0 \end{array}\right]}^{T}$$

Then, the E-vector in b-frame is given by $p_{i}^{b}=C_{m_{i}}^{b}p^{m_{i}}$, where $C_{m_{i}}^{b}$ denotes the installation matrix of the monocular polarization unit, the calibration of which is detailed in Ref [26]. According to the single-scattering Rayleigh based theory, all E-vectors are orthogonal to the solar vector in CPP. The orthogonal relationship is also valid in b-frame, i.e., $p_{i}^{m}\cdot s^{b}=0$. However, a vector that meets the above formula requirement is not just $s^{b}$, but also the anti-the solar vector $-s^{b}$. The ambiguous solar vector is denoted as $s^{b*}$ ($s^{b}$ or $-s^{b}$). Then the nine E-vectors were represented as

(3) $$E=\left[\begin{array}{cccc} p_{1}^{b} & p_{2}^{b} & \cdots & p_{9}^{b} \end{array}\right]$$

As $s^{b*}$ is a unit vector, it can be estimated by minimizing the deviation as

(4) $$L\left(s^{b*},\lambda \right)=s^{b*}{}^{T}EE^{T}s^{b*}-\lambda \left(\left\|s^{b*}\right\|-1\right)$$

$\partial L/\partial s^{b*}=0$ lead to

(5) $$\lambda s^{b*}=EE^{T}s^{b*}$$

Thus, $s^{b*}$ is the eigenvector of the matrix $\left(EE^{T}\right)$ corresponding to the minimum eigenvalue $\lambda_{\min}$ [19,27].

The ambiguousness of the solar vector is eliminated by the obtuse angle between the solar vector and gravity vector as

(6) $$s^{b}=sign\left(\frac{s^{n}\cdot g^{n}}{s^{b}\cdot g^{b}}\right)s^{b*}$$

where, $g^{n}$ is the gravity vector in **n**-frame; $s^{n}$ is the solar vector in n-frame that is obtained by solar ephemeris [28] with local position and time; and $g^{b}$ is gravity vector in b-frame that is calculated by the accelerometer in INS. The black dot (·) means dot product of two vectors.

### 2.2. Integrated Navigation System Modeling

The 15-state vector of INS/POL is given by $X={\left[\begin{array}{lllll} \phi^{T} & \delta v^{T} & \delta P^{T} & \varepsilon^{T} & \nabla^{T} \end{array}\right]}^{T}$, in which $\phi ={\left[\begin{array}{ccc} \phi_{E} & \phi_{N} & \phi_{U} \end{array}\right]}^{T}$ are misalignment angles; $\delta v={\left[\begin{array}{ccc} \delta v_{E} & \delta v_{N} & \delta v_{U} \end{array}\right]}^{T}$ and $\delta P={\left[\begin{array}{ccc} \delta L & \delta \lambda & \delta h \end{array}\right]}^{T}$ are velocity and position errors, respectively; $\varepsilon ={\left[\begin{array}{ccc} \varepsilon_{E} & \varepsilon_{N} & \varepsilon_{U} \end{array}\right]}^{T}$ is gyroscope drift rate; and $\nabla ={\left[\begin{array}{ccc} \nabla_{E} & \nabla_{N} & \nabla_{U} \end{array}\right]}^{T}$ is accelerometer bias. Then, according to the INS error model, the error state equation can be expressed as [29]:

(7) $$\dot{X}=\Phi X+W$$

with,

$$\Phi ={\left[\begin{array}{cc} \Phi_{N} & \Phi_{S}\\ 0_{6\times 9} & 0_{6\times 6} \end{array}\right]}_{15\times 15},\;W={\left[\omega_{\varepsilon_{x}}\;\omega_{\varepsilon_{y}}\;\omega_{\varepsilon_{z}}\;\omega_{\nabla_{x}}\;\omega_{\nabla_{y}}\;\omega_{\nabla_{z}}\;0_{1\times 9}\right]}^{T},$$

where $\Phi_{N}$ is the coefficient matrix related to the three misalignment angles, 3D velocities, and positions of INS. $\Phi_{N}$ and $\Phi_{S}$ [29] are listed in the supplementary file. $\omega_{\varepsilon_{x}}$, $\omega_{\varepsilon_{y}}$, and $\omega_{\varepsilon_{z}}$ are the gyro random walk, and $\omega_{\nabla_{x}}$, $\omega_{\nabla_{y}}$, and $\omega_{\nabla_{z}}$ are the accelerometer random walk.

Considering the misalignment between n-frame and n’-frame, the solar vectors in n’-frame can be expressed in n-frame as

(8) $$s^{n’}=C_{n}^{n’}s^{n}\approx \left[I-\left(\phi \times \right)\right]s^{n}.$$

Then we have

(9) $$s^{n’}-s^{n}=\left(s^{n}\times \right)\phi .$$

Taking into account the measurement noise of the solar vector δs, the calculated solar vector in n’-frame can also be modeled as $s^{n’}=C_{b}^{n’}\left({\tilde{s}}^{b}-\delta s\right)$, where ${\tilde{s}}^{b}$ represents the calculated solar vector obtained from the compound eye polarization sensor by Equations (2)–(6).

Letting $Z=C_{b}^{n’}{\tilde{s}}^{b}-s^{n}$ and $V=C_{b}^{n’}\delta s$, then the POL measurement equation is given as

(10) Z=HX+V,

in which $H=\left[\begin{array}{cc} \left(s^{n}\times \right) & O_{3\times 12} \end{array}\right]$. Thus, the polarization measurement model is established.

## 3. Adaptive Partial Feedback Method

The limitations of the conventional all-feedback method are investigated through a series of simulation experiments. To address the limitations of the all-feedback method for attitude estimation, an attitude angular APF method is proposed. The negative effect of POL measurement is minimized by partial feedback for INS correction. S-B angles are used in the APF method to adaptively adjust the feedback factors.

### 3.1. Performance Analysis of the Conventional All-Feedback Method

In the conventional all-feedback correction, to fuse information from both INS dynamic and POL-based measurement models, the Kalman filter-based method [30] is used in this paper. The continuous integrated system of Equations (6) and (9) are firstly transformed into the following discrete form:

(11) $$X_{k}=\Phi_{k,k-1}X_{k-1}+W_{k-1}$$

(12) $$Z_{k}=H_{k}X_{k}+V_{K}$$

where $W_{k-1}∼N\left(0,Q_{k-1}\right)$ and $V_{K}∼N\left(0,R_{k-1}\right)$ denote the zero-mean white Gaussian system noise and measurement noise, respectively. Then the Kalman filter can be recursively implemented in the following two steps:

- Time Update

(13) $${\hat{X}}_{k/k-1}=\Phi_{k/k-1}X_{k-1}$$

(14) $$P_{k/k-1}=\Phi_{k,k-1}P_{k-1}\Phi_{k,k-1}^{T}+Q_{k-1}$$

- Measurement Update

(15) $${\hat{X}}_{k}=X_{k/k-1}+K_{k}\left(Z_{k}-H_{k}{\hat{X}}_{k/k-1}\right)$$

(16) $$K_{k}=P_{k/k-1}H_{k}^{T}{\left(H_{k}P_{k/k-1}H_{k}^{T}+R_{k}\right)}^{-1}$$

(17) $$P_{k}={\left(P_{k/k-1}^{-1}+P_{k}^{T}R_{k}^{-1}H_{k}\right)}^{-1}$$

where ${\hat{X}}_{k/k-1}$ and $P_{k/k-1}$ represent the predicted state vector and its associated covariance matrix, respectively; ${\hat{X}}_{k}$ and $P_{k}$ denote the estimated state and its associated covariance matrix, respectively; and $K_{k}$ is the Kalman gain.

In the all-feedback method of INS/POL, the first three states estimated by the Kalman filter are completely fed back to INS for the drift errors correction, then the initial values of the states for the next time are set to be zero. The all-feedback strategy is expressed as

(18) $$\left\{\begin{array}{l} \phi_{k}={\hat{\phi }}_{k}\\ {\hat{\phi }}_{k+1}=0_{3\times 3} \end{array}\right.$$

A group of simulation experiments under different $\gamma_{z}$ values were conducted to analyze the inadequacy of the all-feedback strategy of the INS/POL system. In the following section, the analysis of yaw estimation is given for instance. The simulation experiments were designed at a fixed position without translation motion. The $\gamma_{z}$ is adjusted by configuring the solar vector to point in a certain direction in the condition that only the yaw changes. Each experiment in this group lasts 41 min (standing for 1 min then rotating for 40 min). The solar vector is set by adjusting the time. During each experiment period, only the yaw changes continuously from 203.86° to 253.86°, with the horizontal attitude remaining constant. Despite the solar vector changes during the 41 min, this change is negligibly small. The details of simulation assumptions are shown in Table 4. The simulation sensor parameters involved in the simulations are shown in Table 5.

**Figure 4:**
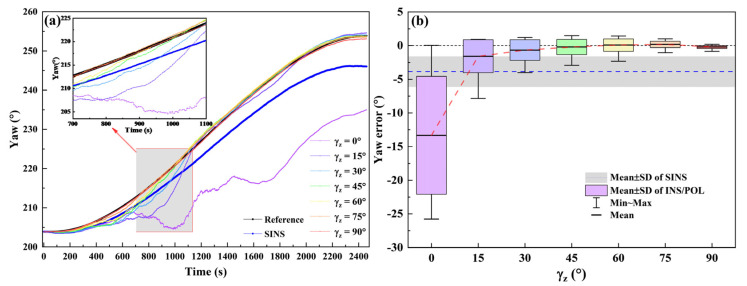
The yaw estimation (**a**) and yaw error (**b**) of SINS and INS/POL for different $\gamma_{z}$. The red dash line is guide to eye.

The simulation results are shown in Figure 4. The estimation accuracy drops greatly as the decreasing of $\gamma_{z}$. In the case of the small $\gamma_{z}$ below 15°, the yaw estimation error is even worse than that of SINS. It indicates that the POL measurement of wide $\gamma_{z}$ can correct INS effectively. The POL measurement of small $\gamma_{z}$, however, has a negative correlation for INS. The results illustrate that the angle $\gamma_{z}$ is able to reflect the correction capability of the POL measurement for INS.

### 3.2. Adaptive Partial Feedback Strategy Based on S-B Angles

**Figure 5:**
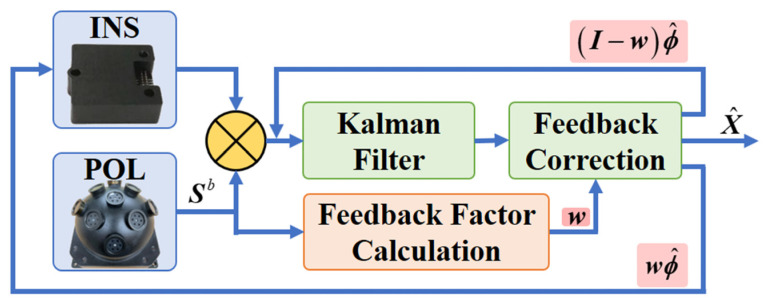
Integration architecture of partial feedback strategy.

To address the low accuracy of attitude estimation of INS/POL based on the all-feedback method on the condition of small S-B angles, the states of misalignment angles estimation are fed back to INS partially. The residual of the state estimation after partial feedback is treated as the initial state value of the filter for the next time. The partial feedback strategy is illustrated in Figure 5.

The feedback factor matrix is represented as w. As the polarization measurement equation is only used for estimating the three misalignment angles, the feedback factor matrix w is set as a 3×3 diagonal matrix. Then the partial feedback strategy can be expressed as

(19) $$\left\{\begin{array}{l} \phi_{k}=w{\hat{\phi }}_{k}\\ {\hat{\phi }}_{k+1}=(I-w){\hat{\phi }}_{k} \end{array}\right.$$

in which $w=\left[\begin{array}{ccc} w_{x} & 0 & 0\\ 0 & w_{y} & 0\\ 0 & 0 & w_{z} \end{array}\right]$, where $w_{x}$, $w_{y}$, and $w_{z}$ are the feedback factors for $\phi_{E}$, $\phi_{N}$, and $\phi_{U}$, respectively. They reflect the degree of correction for three attitude angles, i.e., pitch, roll, and yaw. Actually, the three factors depend on the solar vector measurement noise, the performance of INS, the motion duration in a certain S-B angle, etc. To construct an appropriate form for the factors, a simplified relationship between S-B angles and three factors is considered in this paper:

(20) $$\left\{\begin{array}{l} w_{x}=\frac{1}{2.01}\left(\tanh\left(10\times \left(\sin\gamma_{x}-0.6\right)\right)+1.01\right)\\ w_{y}=\frac{\sin\gamma_{y}+0.01}{1+0.01}\\ w_{z}=\frac{1}{2}\left(\tanh\left(10\times \left(\sin\gamma_{z}-0.3\right)\right)+1\right) \end{array}\right.$$

**Figure 6:**
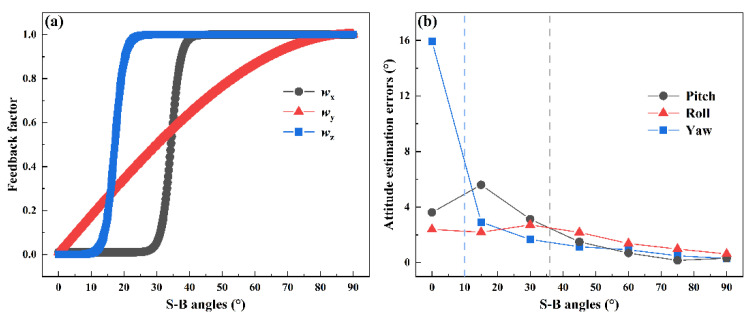
Three attitude feedback factors (**a**) and estimation errors (**b**) against different S-B angles. The blue and gray dash lines in (b) show the transition points of the yaw and pitch estimation error curves against S-B angles, respectively. It can be seen in Figure 6b that the steep fall of the yaw estimation error of the all-feedback method occurs at $\gamma_{z}=15{}^{\circ}$. To further analyze the relationship between the improvement degree and $\gamma_{z}$ in the range around $\gamma_{z}=15{}^{\circ}$, a smaller step in the $\gamma_{z}$ range from 2° to 20° is selected for the simulation condition as shown in Figure 7. The error drops at $\gamma_{z}=4{}^{\circ}$, trends to easing from $\gamma_{z}=8{}^{\circ}$, and becomes smaller than SINS error (4.48°) from $\gamma_{z}=10{}^{\circ}$ (3.30°), and tend to decrease slowly. Thus, when $\gamma_{z}$ is larger than 10°, POL can provide effective correction for INS. Around this point, 0.3 is selected by trial-and-error simulation experiments.

The S-B angles—dependent feedback factors as expressed by Equation (20) are shown in Figure 6a. The three feedback factors are determined by data fitting based on simulation. The results of all-feedback method of different S-B angles show the relationship between the S-B angles and the correction capability of the solar vector for INS. In order to determine the factor $w_{z}$, which directly influences the yaw estimation accuracy, the simulation results of yaw estimation by all-feedback method in different $\gamma_{z}$ are analyzed. As shown in Figure 6a, there is a steep fall of the yaw estimation error depending on $\gamma_{z}$ in the simulation results of INS/POL all-feedback method. A function such as the activation function can be adopted to establish the relationship between the $\gamma_{z}$ and $w_{z}$. Here, the hyperbolic tangent function, y=tanh(x), is selected. The coefficient 10 is introduced to increase the slope near the mutational site. The range of the hyperbolic tangent function is from −1 to 1, but the feedback factor should be from 0 to 1. Then, the constant 1 and coefficient $\frac{1}{2}$ are used to modify the function.

$w_{x}$ is determined in a similar strategy. It differs from the $w_{z}$ in that its constant is 1.01 and the coefficient is $\frac{1}{2.01}$. It is meant to make the minimum value of $w_{x}$ is a small value but not zero. The $w_{y}$ formation is designed as in Equation (11) due to the slow-changing curve of the roll estimation error, which has a linear relationship with $\sin\gamma_{y}$. More details about the simulation results of pitch estimation error based on INS/POL all-feedback under different can be seen in Figure S1 in the supplementary file.

Therefore, the attitude angular adaptive partial feedback model is established to connect the S-B angles and state correction. When the three S-B angles are large enough, the partial feedback factor matrix w approaches a 3×3 identity matrix I, and the AFP is reduced to the conventional all-feedback method.

## 4. Simulation

In this section, some series of simulation experiments are performed to verify the superiority of the APF method. The attitude estimation results of APF and the all-feedback method are compared using a series of simulated static data of different S-B angles. While focusing on the yaw estimation on the condition small $\gamma_{z}$, a dynamic simulation experiment is designed.

### 4.1. Attitude Angle Estimation Analysis Separately with Simulated Static Data

**Figure 8:**
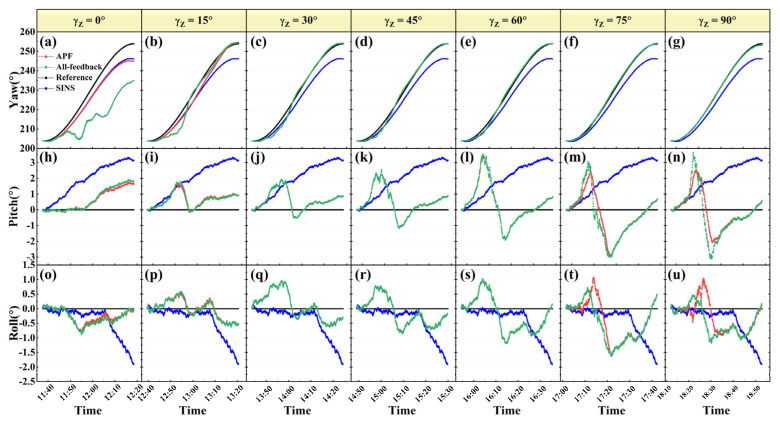
The estimation curves of 3D attitude angles, i.e. yaw (**a**–**g**), pitch (**h**–**n**), and roll (**o**–**u**), for different $\gamma_{z}$.

**Figure 9:**
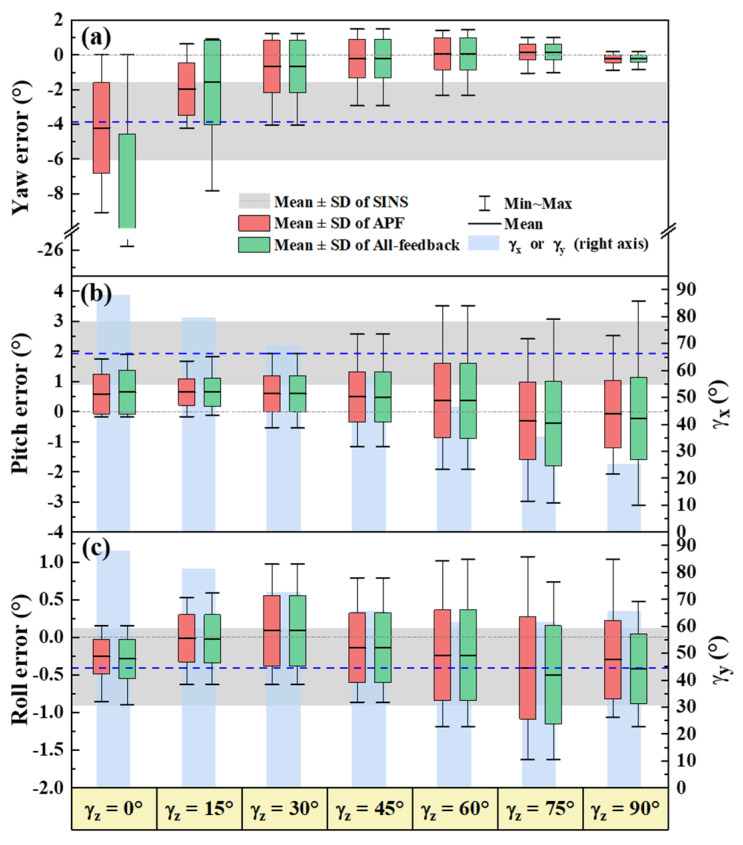
Estimation errors of yaw (**a**), Pitch (**b**), and roll (**c**) for different $\gamma_{z}$.

To evaluate the effectiveness of APF for the three attitude angles separately, three groups of static expe $\gamma_{z}$ riments were designed and carried out. In each group of experiments, one of the three S-B angles is fixed at a certain value by setting the angle between the solar vector and the corresponding platform body axis. The first group of simulation experiments are directing toward evaluating yaw estimation by APF under different $\gamma_{z}$. The simulation condition is the same as the statement in Section 3.1. The yaw and attitude curves and errors are shown in Figure 8 and Figure 9, respectively. Here we compare the results of the proposed APF method with those of the conventional all-feedback method. The top row of Figure 8 shows yaw estimations on the conditions of different $\gamma_{z}$ from 0° to 90°, and the middle and bottom rows illustrate pitch and roll estimations in the corresponding experiment, respectively.

Both the proposed APF and the conventional all-feedback are proved to be capable of estimating 3D attitude correction for INS based on the solar vector-based INS/POL integrated navigation model. It can be seen from the yaw curves that when $\gamma_{z}$ is below 15°, APF can considerably increase the accuracy of yaw estimation compared with the all-feedback method. In particular, when $\gamma_{z}$ is near zero, the root-mean-square errors (RMSE) of the estimation by APF and the all-feedback are 4.95° and 19.94°, respectively. Compared with the all-feedback method, APF improves the accuracy by 68.95%. The yaw estimation error decreases with the increase of $\gamma_{z}$ and tends to be the same as that of the all-feedback method. This similarity is due to the feedback factor of yaw $w_{z}$ approaching 1 gradually as the $\gamma_{z}$ increases. This group of simulation experiments show that, in the condition of small $\gamma_{z}$, APF boosts the yaw estimation accuracy and maintains the estimation accuracies of the horizontal attitude angles at the same time.

**Figure 10:**
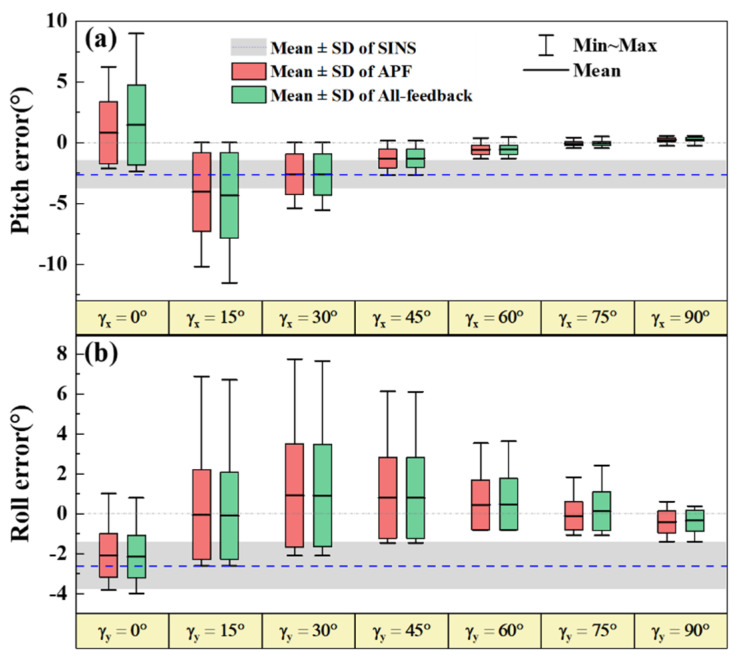
Pitch (**a**) and roll (**b**) estimation errors for different $\gamma_{x}$ and $\gamma_{y}$, respectively.

Despite the performance improvement of APF, the accuracy for small $\gamma_{z}$ is still worse than that for large $\gamma_{z}$. This trend also appears in the pitch and roll estimations as shown in Figure 9b,c. The light blue columns denote the mean values of $\gamma_{x}$ and $\gamma_{y}$ during each experiment. The horizontal attitude angle errors increase as the S-B angles decrease. Another two groups of simulation experiments of pitch and roll estimations when the $\gamma_{x}$ and $\gamma_{y}$ change, respectively, were performed. The results are briefly shown in Figure 10.

In the two groups of simulation experiments, the solar vector is fixed and the S-B angles are set by altering the initial yaw. The details of simulation assumptions and results are shown in Tables S1 and S2, and Figures S2–S5 in the supplementary file, respectively. As shown in Figure 9a, for the pitch estimation results, the accuracy of AFP is better than the all-feedback method, especially for small $\gamma_{x}$. When $\gamma_{x}$ approaches zero, the pitch estimation errors (RMSE) of AFP and the all-feedback are 2.67° and 3.62°. The improvement of AFP reaches 26.24%. Compared with yaw estimation, however, the improvement of APF for pitch is less. While for roll estimation, the improvement of APF is much less. The roll estimation errors of these two methods (AFP is 2.35° and all-feedback method is 2.39°) are almost equivalent. From the results, it is clear that for the small S-B angles, the APF method can improve 3D attitude estimation accuracy with varying percentages. Yaw has the highest improvement, followed by the pitch.

The difference of the improvement degree for the three attitude angles is possibly sought to be the couple between the three attitude angles. The APF method is a simplification by using three partial feedback factors to characterize the correction capability of the solar vector for INS. The complex coupling relationship in the model is ignored.

### 4.2. Yaw Estimation under Small $\gamma_{z}$ with Simulated Dynamic Data

**Figure 11:**
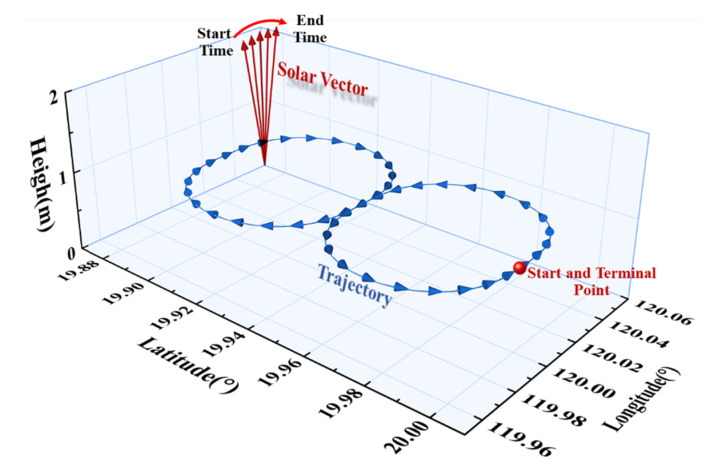
The simulation trajectory.

**Figure 12:**
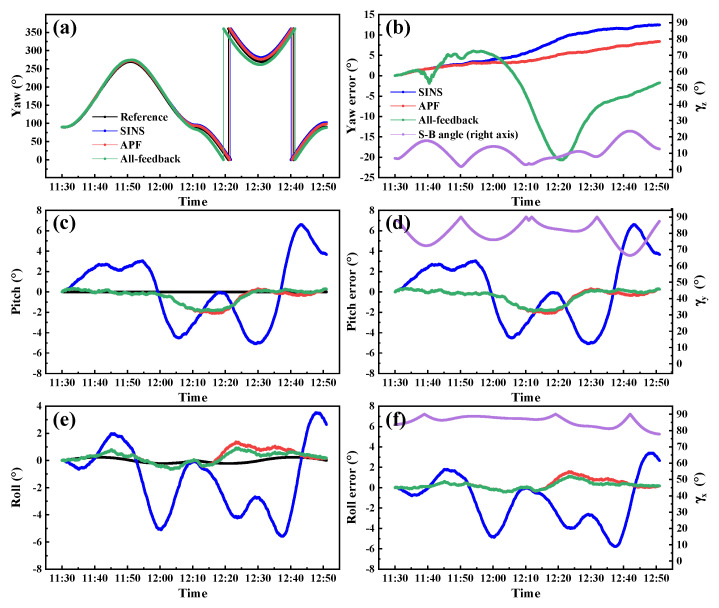
Attitude angle estimation and error curves of dynamic simulation.

It has been shown above that the improvement of yaw estimation by APF is the most significant. Then to further verify the dynamic property of APF for yaw estimation, a dynamic simulation experiment is designed. The sensors’ parameters are the same as the static experiments. A figure-eight trajectory at noon time from 11:30 to 12:51 when the sun is near the zenith is simulated, as shown in Figure 11. As the consequence of the solar motion, $\gamma_{z}$ is not a constant but remains at a small value near 10° during this period, as the purple line in Figure 12b shows.

The yaw, pitch, and roll curves obtained by three methods during the dynamic progress are shown in Figure 12 and the error statistics (RMSE) are listed in Table 6 For small $\gamma_{z}$, the RMSE of the conventional all-feedback method for INS/POL is 8.55°, larger than the 7.72° obtained by SINS, which indicates that it cannot provide reliable yaw estimation in the dynamic experiment. The POL measurement even plays a negative effect for INS in this situation. By contrast, the RMSE of yaw estimation of APF is 4.84°, which is improved by about 43.39%. At the same time, the APF method offers comparable pitch and roll estimation accuracies compared with the all-feedback method.

As can be seen from Figure 12b there is still error accumulated over time for APF. It should be noted that, the original aim of the proposed APF method is to reduce the negative effect of POL measurement for INS/POL system in the condition of small S-B angles, but not able to improve the accuracy of small S-B angles to the same level of large S-B angles.

## 5. Real-World Experiment

**Figure 13:**
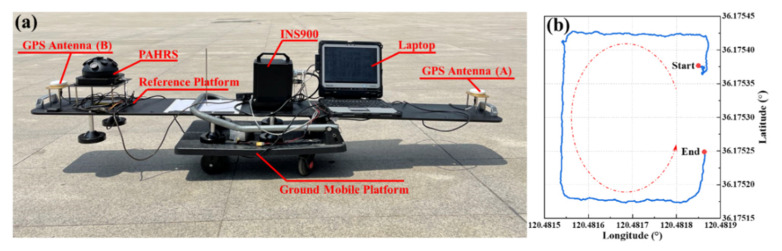
The real-world experiment setup (**a**) and motion trajectory (**b**).

To verify the yaw estimation performance in the realistic scenario by the APF method, an experiment was designed to be performed at noon in summer when the solar altitude is high, which guarantees $\gamma_{z}$ being small enough. The experimental platform was set up, as shown in Figure 13. The reference system is a set of INS/GNSS integrated system (INS900, produced by FOGSINS) equipped with a fiber optic gyroscope and double-antenna GPS. It can provide a high-precision reference with 0.01° for yaw and 0.005° for horizontal attitude. The PAHRS and reference system were mounted on a reference platform. The experiment was performed in a wide-vision square in Licang Qingdao (36.175° N, 120.482° E) from 13:11 to 13:22 (UTC + 8) on July 14, 2021. It was a clear blue sky to obtain reliable celestial polarization information. The ground mobile platform moved around a rectangular trajectory as shown in Figure 13b.

**Figure 14:**
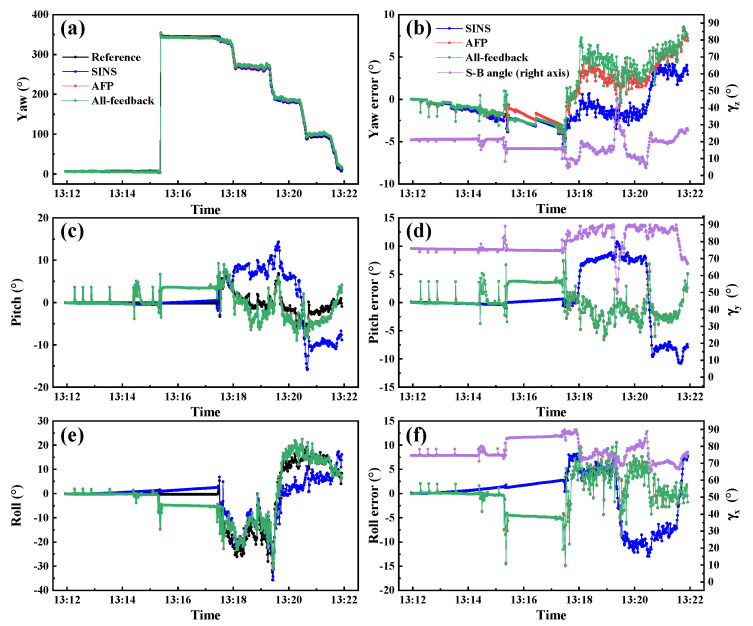
Attitude angle estimation and error curves of real-world experiment.

Figure 14 shows the 3D attitude angles estimation and error curves of the real-world experiment. The error statistics are listed in Table 7. The purple line in Figure 13b shows the $\gamma_{z}$ during the experiment period. It fluctuates around 20°. The results indicate that the yaw estimation accuracy of APF is improved compared with the all-feedback method without losing horizontal attitude angles accuracy. The improvement of yaw estimation accuracy of APF compared with the all-feedback method reaches 18.73% with the RMSE of the two methods being 2.82° and 3.47°, respectively. It can be seen that the percentage improvement achieved by APF for real-world experiment is lower than that for the dynamic simulation experiment, which can be explained by larger $\gamma_{z}$ in real-world experiment. In general, this experiment confirms that the proposed APF method can improve the yaw estimation accuracy in the small $\gamma_{z}$ condition.

## 6. Conclusions

An attitude angular APF method is proposed to reduce the negative effect of polarization measurement with small S-B angles on the INS/POL integrated system. The INS/POL integrated navigation model is established based on the solar vector for 3D attitude determination. In contrast to the conventional all-feedback method, the APF method is able to restrain the negative effect of polarization measurement with small S-B angles by partially feeding back the low-accuracy misalignment angle estimations. To adaptively adjust the correction degree of POL navigation for INS, a feedback factor matrix based on S-B angles was designed. A series of simulation results proved that the APF method can improve 3D attitudes estimation accuracy to varying degrees, in which yaw makes the best. Focusing on the yaw estimation, a real-world experiment was conducted and further verified that the APF can improve yaw estimation accuracy by 18.16% compared with the conventional all-feedback method in the condition that $\gamma_{z}$ is around 20°. This method is a model simplification without considering the coupling of three attitude angles. Future work will optimize the determination strategy of the partial feedback factor matrix by considering the coupling relationship between the three attitude angles.

## Figures and Tables

**Figure 7:**
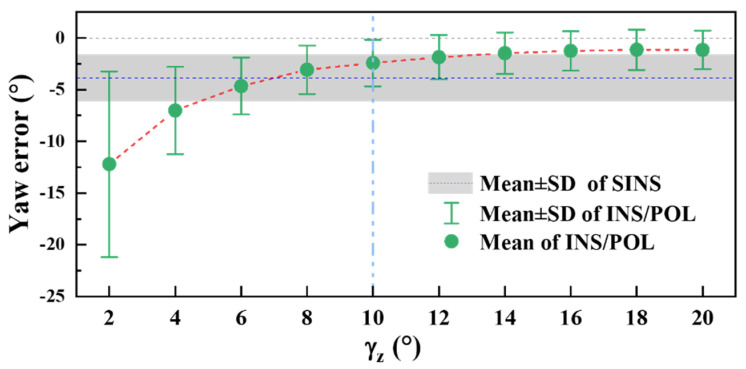
The yaw estimation error of INS/POL for different $\gamma_{z}$ in the range from 2° to 20°. The red dash line is guide to eye and the cyan dash line shows the transition point.

**Table 1:** A review of current INS/POL integrated navigation strategies.

Integrated Strategies	Description
Measurement modeling based on a single E-vector [12,13]	Measurement equation is established by taking advantage of the vertical spatial relationship between E-vector and the solar vector.
Extracting yaw angle from POL measurements	The yaw angle can be calculated from the AoP [11,14] or the solar meridian [15,16] extracted from the AoP pattern image. Then the POL-based yaw is outputted directly or used for measurement modeling with INS.
Based on the solar vector calculated from polarization measurements [17,18]	The solar vector in the body coordinate system is calculated by two or more E-vectors in different view directions [19,20]. Then, the solar vector-based measurement equation can be established.

**Table 2:** Nomenclature.

Acronyms	Definitions
POL	Polarization
INS	Inertial Navigation System
S-B angles	the angles between the Solar-vector and Body-axes of the platform
CPP	Celestial Polarization Pattern
RSN	the Ratio of Signal to Noise
SINS	Strapdown Inertial Navigation System
APF	Adaptive Partial Feedback
IMU	Inertial Measurement Unit
PAHRS	Polarization-based Attitude and Heading Reference System
RMSE	Root-Mean-Square Errors

**Table 3:** The PAHRS features.

Sensors	Specifications
Gyroscope	Bias stability: 2.5°/h
	Random walk: 0.15°/ $\sqrt{h}$
Accelerometer	Bias stability: 3.6 μg
	Random walk: 0.012 m/s/ $\sqrt{h}$
Polarization sensor	AoP accuracy: 0.15° (1 σ)

**Table 4:** Simulation assumptions for evaluating yaw estimation.

Nominal $\gamma_{z}$ /°	Time Interval	Date and Location	Solar Zenith or Real $\gamma_{z}$ Min~Max (Mean)/°
0	11:37:30–12:18:30	1 June 2020 20° N 120° E	2.11~5.25 (3.34)
15	12:39:30–13:20:30		9.93~19.37 (14.64)
30	13:43:30–14:44:30		24.69~34.17 (29.44)
45	14:49:00–15:30:00		39.83~49.26 (44.55)
60	15:54:30–16:38:30		54.86~64.17 (59.52)
75	17:01:30–17:42:30		70.02~79.11 (74.58)
90	18:12:00–18:53:00		85.50~94.41 (89.69)

**Table 5:** Sensor parameters for simulation.

Sensors		Specifications	Frequency
INS	gyroscope	Bias stability: 0.2°/s	20 Hz
		Random walk: 0.01°/ $\sqrt{s}$	
	accelerometer	Bias stability: 1.6 × 10^3^ μg	
		Random walk: 100 μg	
Polarization sensor based solar tracker	Solar zenith	Constant error: 1°	1/3 Hz
		Random error: 0.3°	
	Solar azimuth	Constant error: 0.5°	
		Random error: 0.3°	

**Table 6:** Attitude estimation error statistics of dynamic simulation.

Methods	RMSE (°)		
	Yaw	Pitch	Roll
SINS	7.72	3.24	2.74
APF	4.84	0.86	0.56
All-feedback	8.55	0.80	0.39

**Table 7:** Attitude estimation error statistics of real-world experiment.

Methods	RMSE (°)		
	Yaw	Pitch	Roll
SINS	2.20	5.02	2.15
APF	2.84	2.53	3.81
All-feedback	3.47	2.54	3.79

## Data Availability

Not applicable.

## Supplementary Materials

The following are available online at https://www.mdpi.com/article/10.3390/s22030710/s1, Table S1: Simulation assumptions for evaluating pitch estimation. Table S2: Simulation assumptions for evaluating roll estimation. Figure S1: The pitch estimation error of INS/POL for different $\gamma_{x}$ in the range from 22° to 46°. Figure S2: 3D attitude angles estimation curves for different $\gamma_{x}$. Figure S3: 3D attitude errors for different $\gamma_{x}$. Figure S4: 3D attitude angle estimation curves for different $\gamma_{y}$. Figure S5. 3D attitude errors for different $\gamma_{y}$. More details of the state transition matrix of the INS error model Φ.
