# Supplementary material for: A 3D Attitude Estimation Method Based on Attitude Angular Partial Feedback for Polarization-Based Integrated Navigation System
Source: Sensors (Basel). 2022 Jan 18;22(3):710. doi: 10.3390/s22030710 (PMC8840561; doi:10.3390/s22030710)
Supplement: Supplementary file 1 [file sensors-22-00710-s001.zip › sensors-1495709-Supplementary file updated.pdf]

Supplementary file

Table S1. Simulation assumptions for evaluating pitch estimation

Nominal γ_x/°	Time interval	Date and Location	Initial yaw /°	Real γ_x Min~Max (Mean)/°
0	18:12:00-18:53:00	1/6/2020 20°N 120°E	203.87	0~ 4.87(2.29)
15			218.87	14.03~17.43 (15.35)
30			233.87	28.65~32.14 (30.24)
45			248.87	43.52~47.02 (45.20)
60			263.87	58.44~61.95 (60.18)
75			278.87	73.39~76.90 (75.16)
90			293.87	88.15~90.00 (89.11)

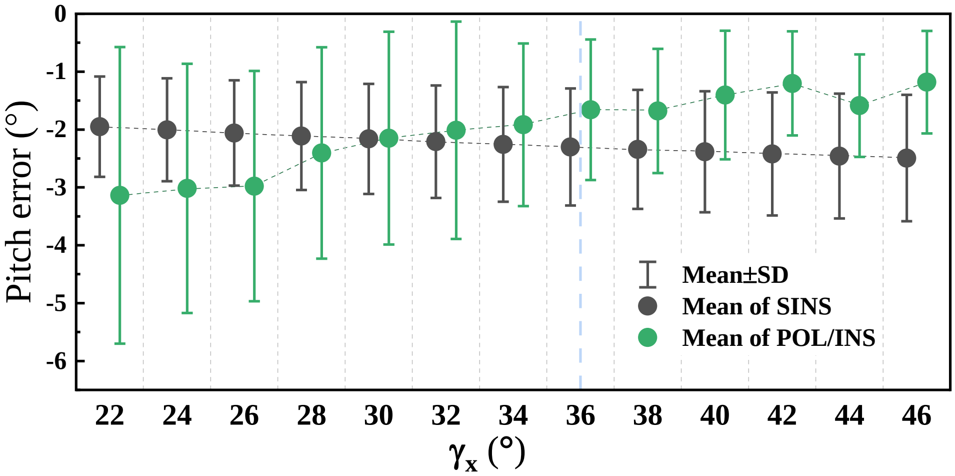

Figure S1. The pitch estimation error of INS/POL for different γ_x in the range from 22° to 46°

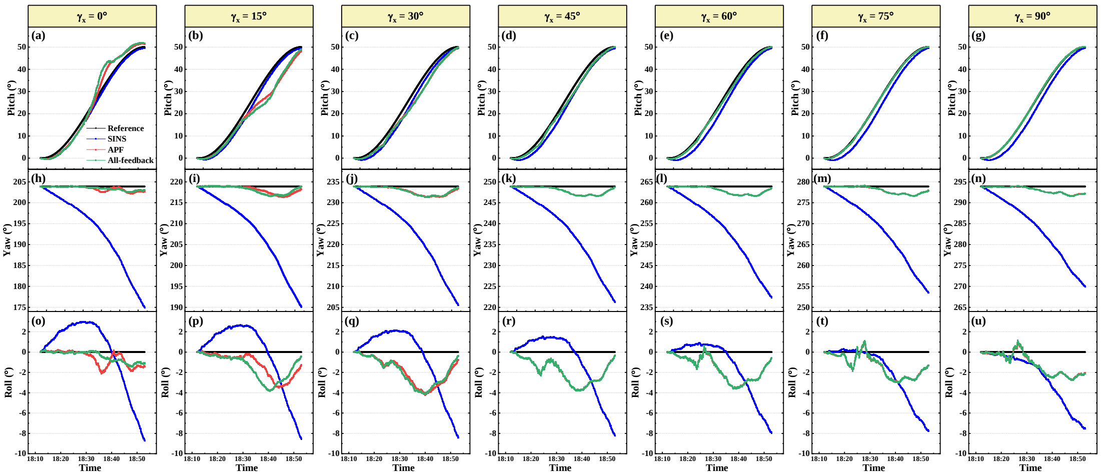

Figure S2. 3D attitude angles estimation curves for different γ_x.

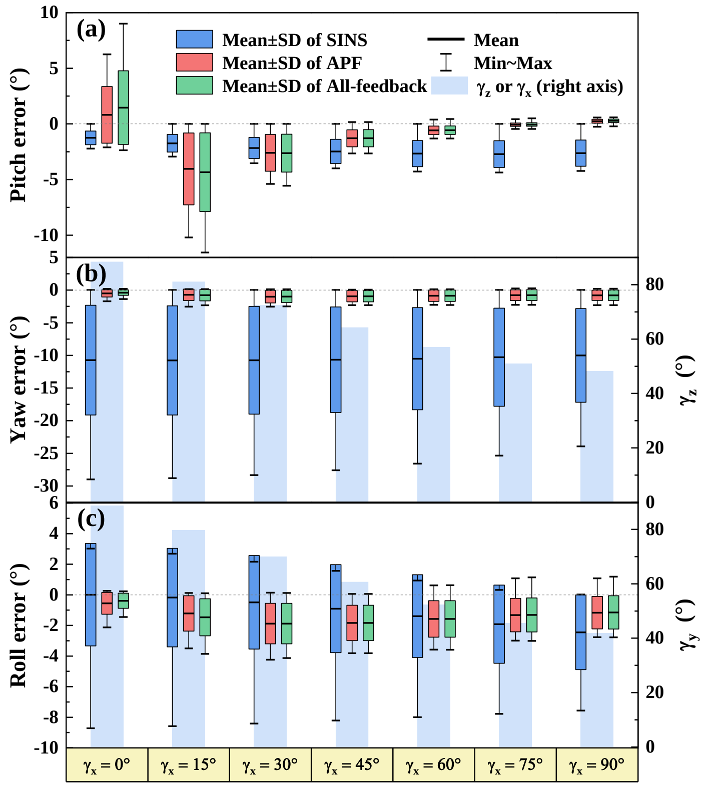

Figure S3. 3D attitude errors for different γ_x.

Table S2. Simulation assumptions for evaluating roll estimation

Nominal γ_y /°	Time interval	Date and Location	Initial yaw /°	Real γ_y Min~Max (Mean)/°
0	18:12:00-18:53:00	1/6/2020 20°N 120°E	293.87	0~4.87(2.29)
15			278.87	13.88~17.21 (15.06)
30			263.87	28.47~31.94 (29.95)
45			248.87	43.33~46.82 (44.91)
60			233.87	58.25~61.75 (59.88)
75			218.87	73.19~76.70 (74.87)
90			203.87	88.15~90.00 (89.11)

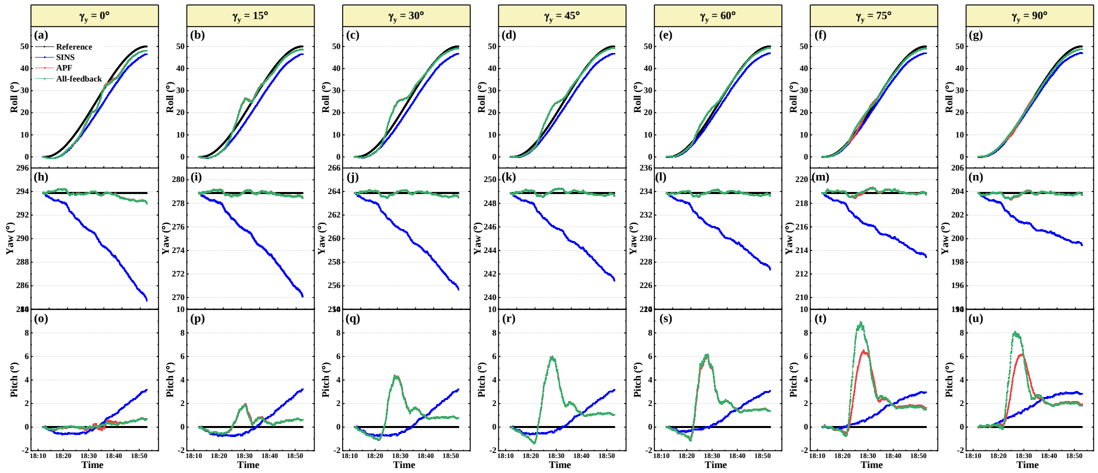

Figure S4. 3D attitude angle estimation curves for different γ_y .

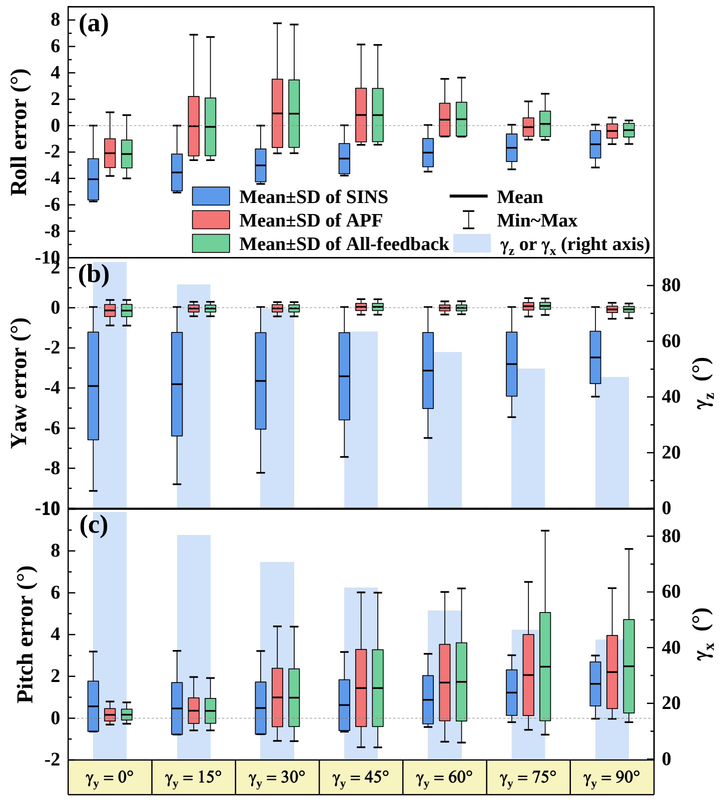

Figure S5. 3D attitude errors for different γ_y .

State-transition matrix $\boldsymbol{\Phi}$:

$$\boldsymbol{\Phi} = \begin{bmatrix} \boldsymbol{\Phi}_N & \boldsymbol{\Phi}_S \\ \mathbf{0}_{6\times 9} & \mathbf{0}_{6\times 6} \end{bmatrix}_{15\times 15}$$

The nonzero elements in $\boldsymbol{\Phi}_N$ are expressed as follows:

$\Phi_N(1,2) = \omega_{ie}\sin L + \frac{v_E}{R_N+h}\tan L$， $\Phi_N(1,3) = -\left(\omega_{ie}\cos L + \frac{v_E}{R_N+h}\right)$,$\Phi_N(1,5) = -\frac{1}{R_M+h}$,

$\Phi_N(1,9) = \frac{v_N}{(R_M+h)^2}$, $\Phi_N(2,1) = -(\omega_{ie}\sin L + \frac{v_E}{R_N+h}\tan L)$, $\Phi_N(2,3) = -\frac{v_N}{R_M+h}$,

$\Phi_N(2,4) = \frac{1}{R_N+h}$, $\Phi_N(2,7) = -\omega_{ie}\sin L$, $\Phi_N(2,9) = -\frac{v_E}{(R_N+h)^2}$, $\Phi_N(3,1) = \omega_{ie}\cos L + \frac{v_E}{R_N+h}$,

$\Phi_N(3,2) = \frac{v_N}{R_M+h}$, $\Phi_N(3,4) = \frac{1}{R_N+h}\tan L$, $\Phi_N(3,7) = \omega_{ie}\cos L + \frac{v_E}{R_N+h}\sec^2 L$, $\Phi_N(4,2) = -f_U$,

$\Phi_N(3,9) = -\frac{v_E}{(R_N+h)^2}\tan L$, $\Phi_N(4,3) = f_N$, $\Phi_N(4,4) = \frac{v_N}{R_M+h}\tan L - \frac{v_U}{R_M+h}$,

$\Phi_N(4,5) = 2\omega_{ie}\sin L + \frac{v_E}{R_N+h}\tan L$, $\Phi_N(4,6) = -2\omega_{ie}\cos L - \frac{v_E}{R_N+h}$,

$\Phi_N(4,7) = 2\omega_{ie}v_N\cos L + \frac{v_E v_N}{R_N+h}\sec^2 L + 2\omega_{ie}v_U\sin L$, $\Phi_N(4,9) = \frac{v_E v_U - v_E v_N \tan L}{(R_N+h)^2}$, $\Phi_N(5,1) = f_U$,

$\Phi_N(5,3) = -f_E$, $\Phi_N(5,5) = -\frac{v_U}{R_M+h}$, $\Phi_N(5,4) = -(2\omega_{ie}\sin L + \frac{v_E}{R_N+h}\tan L)$, $\Phi_N(5,6) = -\frac{v_N}{R_M+h}$,

$\Phi_N(5,7) = -(2\omega_{ie}v_E\cos L + \frac{v_E^2}{R_N+h}\sec^2 L)v_E$, $\Phi_N(5,9) = \frac{v_N v_U + v_E^2\tan L}{(R_N+h)^2}$, $\Phi_N(6,1) = -f_N$,

$\Phi_N(6,2) = -f_E$, $\Phi_N(6,4) = 2(\omega_{ie}\cos L + \frac{v_E}{R_N+h})$, $\Phi_N(6,5) = \frac{2v_N}{R_M+h}$, $\Phi_N(6,7) = -2\omega_{ie}v_E\sin L$,

$\Phi_N(6,9) = -\frac{v_E^2 + v_N^2}{(R_N+h)^2}$, $\Phi_N(7,5) = \frac{1}{R_M+h}$, $\Phi_N(7,9) = -\frac{V_N}{(R_M+h)^2}$, $\Phi_N(8,4) = \frac{\sec L}{R_N+h}$,

$\Phi_N(8,7) = \frac{v_E}{R_N+h}\sec L\tan L$, $\Phi_N(8,9) = -\frac{v_E}{(R_N+h)^2}\sec L$, $\Phi_N(9,6) = 1$

where， R_M is the radius of curvature of meridian， R_N is the radius of curvature in prime vertical， ω_{ie} is the earth's rotation angular velocity， L is the local longitude， h is the height of vehicle， v_E, v_N, v_U are 3D velocities and f_E, f_N, f_U are the outputs of tri-axis accelerometer.

Φ_S is expressed as

$$\Phi_s = \begin{bmatrix} C_b^n & 0_{3\times3} \\ 0_{3\times3} & C_b^n \\ 0_{3\times3} & 0_{3\times3} \end{bmatrix}_{9\times6}$$
